# Characterization of Stem-Like Cells in Mucoepidermoid Tracheal Paediatric Tumor

**DOI:** 10.1371/journal.pone.0107712

**Published:** 2014-09-17

**Authors:** Mei Ling Lim, Brandon Nick Sern Ooi, Philipp Jungebluth, Sebastian Sjöqvist, Isabell Hultman, Greg Lemon, Ylva Gustafsson, Jurate Asmundsson, Silvia Baiguera, Iyadh Douagi, Irina Gilevich, Alina Popova, Johannes Cornelius Haag, Antonio Beltrán Rodríguez, Jianri Lim, Agne Liedén, Magnus Nordenskjöld, Evren Alici, Duncan Baker, Christian Unger, Tom Luedde, Ivan Vassiliev, Jose Inzunza, Lars Ährlund-Richter, Paolo Macchiarini

**Affiliations:** 1 Advanced Center for Translational Regenerative Medicine, Department for Clinical Science, Intervention and Technology, Division of Ear, Nose, Throat, Karolinska Institutet, Stockholm, Sweden; 2 School of Applied Science, Republic Polytechnic, Singapore; 3 Department of Women's and Children's Health, Karolinska Institutet, Stockholm, Sweden; 4 Department of Oncology and Pathology, Karolinska University Hospital, Stockholm, Sweden; 5 Center for Hematology and Regenerative Medicine, Department of Medicine, Karolinska Institutet, Stockholm, Sweden; 6 International Scientific-Research Clinical and Educational Center of Regenerative Medicine, Kuban State Medical University, Krasnodar, Russian Federation; 7 Department of Clinical Genetics, Karolinska University Hospital, Stockholm, Sweden; 8 Department of Molecular Medicine and Surgery and Center for Molecular Medicine, Karolinska Institutet, Stockholm, Sweden; 9 Department of Biomedical Sciences, University of Sheffield, Sheffield, United Kingdom; 10 Department of Medicine III, University Hospital RWTH Aachen, Germany; 11 Robinson Institute, Center for Stem Cell Research, The University of Adelaide, Adelaide, Australia; 12 Department of Biosciences and Nutrition, Karolinska Institutet, Karolinska University Hospital, Huddinge, Stockholm, Sweden; University Hospital of Modena and Reggio Emilia, Italy

## Abstract

Stem cells contribute to regeneration of tissues and organs. Cells with stem cell-like properties have been identified in tumors from a variety of origins, but to our knowledge there are yet no reports on tumor-related stem cells in the human upper respiratory tract. In the present study, we show that a tracheal mucoepidermoid tumor biopsy obtained from a 6 year-old patient contained a subpopulation of cells with morphology, clonogenicity and surface markers that overlapped with bone marrow mesenchymal stromal cells (BM-MSCs). These cells, designated as MEi (mesenchymal stem cell-like mucoepidermoid tumor) cells, could be differentiated towards mesenchymal lineages both with and without induction, and formed spheroids *in vitro*. The MEi cells shared several multipotent characteristics with BM-MSCs. However, they displayed differences to BM-MSCs in growth kinectics and gene expression profiles relating to cancer pathways and tube development. Despite this, the MEi cells did not possess *in vivo* tumor-initiating capacity, as proven by the absence of growth *in situ* after localized injection in immunocompromised mice. Our results provide an initial characterization of benign tracheal cancer-derived niche cells. We believe that this report could be of importance to further understand tracheal cancer initiation and progression as well as therapeutic development.

## Introduction

Primary tracheal tumors are very rare, representing only up to 0.2% of all respiratory malignancies [1]–[3]. This is particularly true in the paediatric population. The most common tracheal neoplasm reported in children is mucoepidermoid carcinoma, a salivary gland-type cancer [4], [5]. The mucoepidermoid tumors are histologically heterogenous low-grade tumors that grow locally, without metastasis [3], [6], [7]. It is usually identified by a characteristic translocation/fusion transcript at t(11;19) [8]. Due to their rarity, the characteristics and biology of these neoplasms remain poorly understood. However, it has been proposed that tracheal tumors may originate from niche cells that reside in the respiratory epithelium, glands or mesenchymal niches. These could be either a population of tissue stem cells, transformed progenitor cells or cancer stem cells (CSCs) [9]–[11].

Normal stem cells and tumorigenic cells share many resemblances with regard to gene expression profiles, morphology and both have extensive proliferative potential with the ability to give rise to new (normal or abnormal) tissues [12]–[14]. The growth of solid cancers has been suggested to be driven by what has been generally termed ‘cancer stem cells’ (CSCs), reported from malignant tumors of various tissues such as lung [15]–[18], pancreas [19]–[21], prostate [22]–[25], colon [26] and breast [27]. Normal stem cells and CSCs show also similarities with regard to their dependencies on sonic hedgehog (Shh) [28], [29], Notch [9] and Wnt [30], [31] pathways.

A presence of stem-like cells detected in also benign tumors, as shown in the present paper, is in accordance with a previous report by Xu and colleagues studying pituitary adenoma [32]. However, stem cells have so far not been demonstrated in transformed tissues from the human upper respiratory tract. We here identified and characterized the expanded primary cultures from a benign paediatric mucoepidermoid tracheal tumor.

## Materials and Methods

### Ethics statement

#### Animal experiments

Animal experimentation was performed according to ethical permission numbers N173/10 (Stockholm Northern Animal Review Board) and S180/12 (Stockholm South Ethical Committee). All animals were treated in compliance with the “Principles of laboratory animal care” formulated by the National Society for Medical Research and the “Guide for the care and use of laboratory animals” prepared by the Institute of Laboratory Animal Resources, National Research Council, and published by the National Academy Press, revised 1996. All surgery was performed under anesthesia, and all efforts were made to minimize animal pain and suffering.

#### Patient sample

The Stockholm Regional Ethical Review Board has approved the study to collect patient material according to ethical permission numbers 2008 307-31 and 2012 2163-311 with written informed parent's consent to publish. All clinical research was conducted according to the principles expressed in the Declaration of Helsinki. A tracheal sample was obtained from a 6-year-old female patient. She was surgically treated for a diagnosis of primary mucoepidermoid tumor, and underwent subtotal tracheotomy at Karolinska University Hospital, Stockholm, Sweden. Half of the tissue was fixed and paraffin-embedded for pathological analyses, and half processed for cellular and molecular analyses.

### Tracheal patient pathology

The tissue was fixed, paraffin-embedded and sectioned at 5 µm, de-paraffinized and stained for the following: Haematoxylin Eosin (HE) (Histolab, Sweden), periodic acid-Schiff stain (DAKO, Denmark), Ki67 (DAKO), Muc-1 (BD Biosciences, CA, USA), cytokeratin markers (Ck) MNF116 (DAKO), carcino-embryonic antigen (CEA) (DAKO) and androgen receptor (Ventana, Switzerland). Three pathologists determined the proliferation index independently by manually counting the number of proliferative cells present in 10 fields at 40× magnification. Total RNA was purified from paraffin sections with a QIAamp RNeasy Kit and was processed according to manufacturer's instructions (Qiagen, Germany). cDNA was obtained with a high-capacity cDNA reverse transcription kit (Applied Biosystems, CA, USA). The samples were run on *7000* Fast Real-Time PCR System (Applied Biosystems) in duplicates with TaqMan probes for detection of fusion transcripts.

### Isolation, expansion and maintenance of trachea tumor cells

Tumor tissue was manually minced with a scalpel followed by enzymatic digestion at 37°C; 5% CO_2_ for 1.5 hours in 24 U/ml Dispase and 1% Collagenase Type 1a (all from Invitrogen, Life Technologies, Sweden) in Hank's Balanced Salt Solution. The collagenase activity was discontinued on ice and the cell suspension was then strained with a 70 µm cell strainer (BD Biosciences, Sweden), centrifuged for 6 minutes at 600 *g* and finally seeded into a 75 cm^2^ flask with tracheal culture medium (see below). Fifty percent of the culture medium was changed after 4 days and subsequently the medium was changed completely every three days. When cells reached 90% confluence, cells were trypsinized for 5 minutes at 37°C with TryPLe Select (Invitrogen, Life Technologies), collected and centrifuged for 5 minutes at 300 *g* and cell pellet was subcultured at a 1∶3 ratio.

### Culture medium of tracheal tumor cells

Cells were cultured and expanded in Dulbecco's modified Eagle's medium with low glucose (DMEM), 10% fetal bovine serum (FBS) and 1% antibiotics (penicillin/streptomycin) and antimycotics (amphotericin B) (all from Invitrogen, Life Technologies).

### Cell sorting trachea tumor cells by FACS

Trachea cells at passage 12 were trypsinized and resuspended in FACS (Fluorescence Activated Cell Sorting) buffer (Dulbecco Phosphate buffered solution without calcium and magnesium, 2% Fetal Bovine Serum and 1 mM EDTA) and strained through a 70 µm cell strainer to avoid aggregates. Single cells suspensions were incubated with previously titrated antibody mix: CD105, CD90, CD73, CD44, CD31, CD45, CD34, CD11b, HLA-DR and CD14 (all from BD Biosciences) and incubated for 20 minutes at room temperature. After washing, the cells were resuspended in FACS buffer at a concentration of 20×10^6^/ml. Cells were sorted with an Aria III (BD Biosciences) equipped with 405 nm, 488 nm, 561 nm and 633 nm lasers. Cells were recovered and grown to confluence in DMEM, 20% FBS and 1% antibiotics (penicillin/streptomycin). The post-sorted mucoepidermoid tumor cells will hereafter be referred as MEi (MSC-like mucoepidermoid tumor) cells, p9+1 and p12+1. MEi cells from passages p9+2 to p9+6, p12+2 to p12+6 are used in this study.

### FACS isolation and analysis of human BM-MSCs

Human BM-MSCs were sorted, expanded to passage 6 and used as a positive control. BM-MSCs were isolated by multi-color FACS as described previously [33]. Briefly, mononuclear cells from BM aspirates of healthy adult volunteers were isolated by Ficoll-Hypaque (Lymphoprep, Axis-Shield PoC AS, Norway) density centrifugation. The CD45^−^CD235^−^ cells were enriched by negative selection using CD45 and CD235 microbeads and magnetic-activated cell sorting (MACS, Miltenyi Biotec, Germany). The cells were stained with anti-human CD271 (Biolegend, CA, USA), CD146 (Biolegend), CD45− and Glycophorin A/CD235 (eBioscience, CA, USA). Dead cells were excluded by propidium iodide staining (Sigma-Aldrich, Sweden). The CD44+ MSCs were sorted on FACSAria II Sorp (BD Biosciences).

### Immunocytochemistry staining

Lineage tracing markers were used to determine tracheal mucoepidermoid tumor origin at passage 3. To further determine the differentiation potential of MEi (MSC-like mucoepidermoid tumor) cells in comparison to BM-MSCs, cells were induced and stained for endoderm and ectoderm lineage markers. Cells were fixed with 4% formalin (Histolab) at room temperature for 10 minutes and were washed three times. Cells were blocked with 5% FBS (Invitrogen, Life Technologies) and 0.1% Triton X (Sigma-Aldrich) in DPBS for 1 hour at room temperature on a rocking platform. The cells were stained with primary antibodies on a rocking platform overnight at 4°C. Trachea tumor cells before and after sorting were stained with mouse monoclonal to MUC 1 (1∶50) (ab70475, Abcam, UK) and rabbit polyclonal to MAML2 (1∶50) (ab90592, Abcam). The cells were washed with DPBS with 0.1% Tween 20 (Sigma-Aldrich). The corresponding secondary antibodies, Alexa 488 goat anti rabbit (1∶2000) (A11008, Life technologies), and Alexa 594 goat anti mouse (1∶2000) (A11032, Life technologies) were incubated on a rocking platform at room temperature for 1 hour.

MEi cells were stained with primary antibodies overnight at 4°C for endoderm; rabbit monoclonal to GATA6 (1∶1600) (#5851, Cell Signaling) and ectoderm; rabbit monoclonal to β III tubulin (1∶100) (#5568, Cell Signaling, USA) markers. Cells were washed with DPBS with 0.1% Tween 20 (Sigma-Aldrich). The corresponding secondary antibody, Alexa 488 goat anti rabbit (1∶500) (A11008, Life technologies) was incubated on a rocking platform at room temperature for 1 hour. After secondary antibody application, cells were washed with DPBS with 0.1% Tween 20 and were counterstained with SlowFade Gold antifade mountant with nuclear marker 4′,6-diamidino-2-phenylindole (DAPI) (Molecular Probes, Life Technologies). Cells were visualized and imaged under an inverted microscope (Olympus IX70, Japan). Negative controls for each secondary antibody were performed without the addition of primary antibody, and no non-specific binding was detected. The number of positive MUC 1 and MAML2 and DAPI stained cells was quantified by manually counting in 3 fields at ×20 magnification. Results are presented in percentages.

### Mathematical modeling of *in vitro* growth of trachea tumor cells

A mathematical modeling was designed to calculate the doubling times of cells, and to estimate the number of MEi (MSC-like mucoepidermoid tumor) cells as a proportion of the total number of adherent cells from the original tumor tissue.

#### Estimation of cell doubling times

Unsorted tumor cells, MEi cells and BM-MSCs were initially seeded in triplicates at cell densities of 10^3^/0.32 cm^2^. Dead cells were identified by trypan blue (Sigma-Aldrich) and were manually counted in triplicates with a Bürker counting chamber (Marienfeld, Germany). The estimates of the doubling times were calculated by assuming exponential growth in cell numbers, hence N(t) = N_0_ exp (kt) (1) where N is the number of cells in culture after a time t, and N_0_ and k are regression parameters corresponding respectively to the number of cells in the culture at t = 0 and the cell proliferation rate. Using least squares minimization, equation (1) was fitted to time courses of the cell numbers from each well, giving a total of 9 pairs of values of N_0_ and k. The mean proliferation rates for unsorted tumor cells, MEi cells, and BM-MSCs were found to be k = 0.2243, 0.2502, and −0.0182 day^−1^ respectively. Using the fitted value of k for each corresponding well, the doubling times were calculated using τ = ln 2/k. The mean and uncorrected standard deviations of doubling times were found to be τ = 3.3±0.8 days for unsorted tumor cells, τ = 2.8±0.4 days for MEi cells, and τ = 63±132 days for BM-MSCs.

#### Estimation of the proportion of MEi (MSC-like mucoepidermoid tumor) cells that reside at tumor tissue

It was found that after t = 21 days (3 weeks) of culturing the adherent cells from the tumor tissue, there were a total of 3.25×10^6^ cells and, of the 3×10^6^ cells that were analyzed, there were 903.7×10^3^ MEi cells. Therefore, the total number of MEi cells at the end of the culture period was 903.7×10^3^×3.25/3≈979×10^3^.

To calculate the number of cells at the initial of the culture (passage 0), equation (1) was rearranged to give N_0_ in terms of N, k and t: N_0_ = N exp (-kt). (2) For unsorted tumor cells, applying equation (2) with N = 3.25×10^6^, k = 0.224 and t = 21 gave N_0_ = 29.2×10^3^ as the number of cells that were present at the initial culture period. For MEi cells, applying (2) with N = 979×10^3^, k = 0.250 and t = 21 gave N_0_ = 5.1×10^3^ as the number of cells that were present at the beginning of the culture. Hence the proportion of adherent cells that were MEi cells in the tumour tissue was estimated to be 17.5%.

### Colony forming fibroblast (CFU-F) assay

MEi (MSC-like mucoepidermoid tumor) cells and BM-MSCs were cultured for 13 days *in vitro* at a cell density of 10^4^/9.60 cm^2^. Cells were fixed for 10 min at room temperature in 4% formalin (Histolab). Cells were washed three times and 0.06% Giemsa staining (Sigma-Aldrich) was incubated for 5 minutes at room temperature. The staining was gently rinsed with distilled water and air-dried. A colony was defined as a stained cluster of approximately ≥50 cells. The colonies were quantified under brightfield microscopy (Olympus IX70, Japan).

### Suspension cultures for production of spheroids

MEi cells were trypsinized and either seeded onto ultra-low attachment surfaces (Corning, NY, USA) in culture media at a cell density of 2×10^5^/cm^2^ or alternatively placed in hanging drop cultures of 15 to 20 cells per drop on the lid of a Petri dish (BD Biosciences) in a static culture condition. The suspension culture media was refreshed every third day and the hanging drops were topped up with more culture media every third day.

### Spheroid culture medium

Dulbecco's modified Eagle's medium, low glucose was supplemented with 20% fetal bovine serum (FBS), 1×penicillin-streptomycin, 1×GlutaMAX and 1×non-essential amino acid solution and 0.5 mM 2-mercaptoethanol (all from Invitrogen, Life Technologies).

### Directed and spontaneous differentiation and evaluation of spheroid and monolayer cultures

Day 10 spheroids from MEi cells were transferred from ultra low attachment suspension culture and plated onto non-coated BD Falcon center-well IVF dish (BD Biosciences). The spheroid outgrowths were either differentiated under two conditions: with or without differentiation media. The outgrowths were directly differentiated with STEMPRO osteogenic, adipogenic and chondrogenic differentiation kits (Invitrogen, Life Technologies) and analyzed at day 12 and 24. The outgrowths (both directed and spontaneous differentiation) were stained with Toluidine Blue, Alizarin Red and Oil Red, respectively (all Sigma-Aldrich, MO, USA) to confirm mesenchymal trilineage differentiation. Outgrowths without differentiation media were further confirmed by flow cytometry at day 25 for a panel of mesenchymal stromal cell markers.

Monolayer cultures from MEi cells and BM-MSC were seeded for 48 hours at a cell density of 10^3^ cells in 4 well BD Falcon CultureSlides (BD Biosciences) and were induced for 7 days with 100 ng/ml BMP4 (endoderm) (R&D systems, Sweden) or 100 ng/ml Retinoic acid (mesoderm) (Sigma-Aldrich). Cell culture media was refreshed every second day [34].

### Total RNA extraction from cells

Total RNA was isolated from the freshly isolated and cultured human cells using a commercially available RNeasy Mini Kit (Qiagen). RNA extraction was processed according to the manufacturer's instructions.

### MicroRNA isolation and purification from cells

MicroRNA from BM-MSCs, unsorted tumor cells, sorted (MEi cells) and spontaneously differentiated spheroid outgrowths were isolated by phenol/chloroform method. Briefly, 800 µL phenol (Qiazol, Sweden) and 200 µL chloroform were added and mixed vigorously for 15 seconds followed by incubation at room temperature for 10 minutes. The samples were centrifuged for 30 minutes at 12,000 *g* until complete phase separation. The aqueous phase (with total RNA) was precipitated with 500 µL 100% isopropanol and 2 µL glycogen (Fermentas, Germany) overnight at −20°C. Samples were centrifuged at 4°C for 15 minutes at 12,000 *g* and obtained pellets were washed once with 70% ethanol. The precipitated RNA was re-suspended in 30 µL RNase free water (Ambion, Austin, TX). RNA quantity and quality was assessed using a NanoDrop spectrophotometer (NanoDrop, Wilmington, DE, USA) and a smallRNA assay for Agilent's Bioanalyzer (Agilent Technologies, Germany).

### cDNA preparation

The Affymetrix Gene Chip WT Sense Target Labeling and Control Reagents kit was used for preparation of cDNA from 100 ng of total RNA according to the manufacturer's protocol. Briefly, total RNA was first reverse transcribed using a T7-Oligo (dT) Promoter Primer in the first-strand cDNA synthesis reaction. Following RNase H-mediated second-strand cDNA synthesis, the double-stranded cDNA served as a template in the subsequent *in vitro* transcription (IVT) reaction carried out in the presence of T7 RNA Polymerase. cRNA was purified using the Nucleic Acid Binding Beads and quantified by UV absorbance at 260 nm on the ND1000 spectrophotometer (Thermo Scientific, MA, USA). Sense strand cDNA was synthesized by the reverse transcription of 10 µg of cRNA using random primers. The cRNA template was hydrolyzed using RNase H and single stranded cDNA purified and quantified using the same methods described above.

### Microarray hybridization

cDNA was fragmented and labeled using the GeneChip WT Terminal Labeling Kit. The Affymetrix Gene Chip Human Gene 1.1 ST Array plates were used for hybridization. Array hybridization was done at 45°C, 60 rpm for 17 hours. Chips were washed, stained and scanned on a Gene Titan Instrument according to the manufacturer's recommendations.

### Microarray data analysis

Signal intensities from raw CEL files were extracted, normalized and summarized by using the robust multi-array average (RMA) function implemented in the R Bioconductor software. Affymetrix control probe and probe sets with little variations across samples and no Entrez Gene identifiers were filtered out. Filtered probe sets were used to group samples by Principal Component Analysis (PCA) and the top 100 probe sets with the highest interquartile range were used to group the samples by hierarchical clustering. To identify genes that were significantly different between the tumor cells and BM-MSC, a modified *t*-test incorporating the Benjamini-Hochberg multiple hypotheses correction technique was used [35]. A total of 454 differentially expressed genes with fold change greater than 4 were processed using the Database for Annotation, Visualization and Integrated Discovery (DAVID) Gene Annotation Tool [36] to identify biological differences between the two cell types. Microarray data are available in the ArrayExpress database (www.ebi.ac.uk/arrayexpress) under accession number E-MTAB-2093

### Karyotype analysis

Slides for metaphase G-banding were prepared using standard techniques. Cells were treated with 0.1 µg/ml Colcemid (Invitrogen, Life Technologies) for up to 4 hours, followed by dissociation with trypsin/versene. The cells were pelleted via centrifugation and resuspended in pre-warmed 0.0375 M KCl hypotonic solution and incubated for 10 minutes. Following centrifugation the cells were resuspended in fixative (3∶1 methanol∶acetic acid). One drop of suspension was dropped onto a glass microscope slide (Sigma-Aldrich).

### G-banding

G-banding was performed by exposure to trypsin for 25 seconds and stained with 4∶1 Gurr's/Leishmann's stain (Sigma-Aldrich) for 2 minutes. G-banded slides were scanned, metaphases captured and analysed using a Cytovision GSL-120 (Leica Microsystems, Switzerland) image analysis system. A minimum of 5 metaphase spreads were analyzed and a further 45 counted and scored. A health professional council registered Clinical Scientist in Clinical Pathology Accredited laboratory performed the analyses.

### Molecular karyotyping

For Array Comparative Genomic analysis, an 180 K oligonucleotide microarray with even whole genome coverage and median probe spacing of approximately 16 kb was used (Oxford Gene Technology, UK). Genomic DNA isolated from the cells of primary tracheal tumor patient and a sex-matched pooled reference DNA isolated from healthy controls (Promega, WI, USA) were labeled with Cy3 and Cy5 respectively (Enzo Life Sciences, NY, USA). Hybridization and slide washing (Oligo aCGH/ChIP-on-Chip Wash Buffer Kit, Agilent Technologies, DE, USA) were performed according to the manufacturers' recommendations. Scanning of the array slide was performed on a microarray scanner with 3 µm resolution and initial data analysis was performed with the Feature Extraction software v 10.7.3.1 (Agilent Technologies) followed by analysis with the CytoSure Interpret Software v 4.3 (Oxford Gene Technology).

### Analysis of *in vivo* growth

Trachea tumor cells sorted for MSC markers were either injected as dissociated cells (1×10^6^/mouse) or as day 10 spheroids (20/mouse). Cells were injected into 6 to 8 weeks old male SCID/Beige mice (C.B.-17/GbmsTac-scid-bgDF N7; M&B; C.B-17/scid-beige, Taconic) either under the testes capsule (n = 6) or subcutaneously (n = 5). In the group with subcutaneous injection, growth factor reduced Matrigel (BD Biosciences) was used as vehicle. The animals were euthanized at 8 weeks after inoculation. For the microscopic analysis, testis and fibrous/fat tissue at the site of subcutaneous injections were excised and fixed overnight at 4°C in 4% paraformaldehyde (Histolab), dehydrated through a graded series of alcohol to xylene, embedded in paraffin, and serially sectioned at 5 µm thickness.

Sections were subjected to standard HE staining for basic histological orientation. For the identification of human cells, Fluorescent In Situ Hybridization (FISH) was performed using a mixed probe against the human X-chromosome (red) and Y- chromosome (green) (CEP XY; Vysis Inc, IL, USA), as previously described [37].

### Statistical analysis

Data analyses were performed using Student t-tests and preparation of graphs, and statistical comparisons were undertaken with Prism software (GraphPad Prism version 5.0, MAC version). Statistical significance was accepted at p<0.05.

## Results

### Pathological evaluation was consistent with a diagnosis of mucoepidermoid cancer

**Figure 1:**
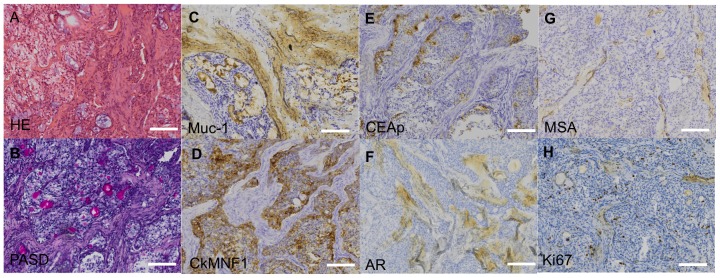
Histological and pathological analyses of a mucoepidermoid tumor. Haematoxylin and Eosin staining provides visualization of the excised tracheal mucoepidermoid tumor biopsy (a), periodic acid-Schiff stain identifies mucins and glycoproteins in magenta (b), Mucin 1, Muc-1 identifies mucin-producing cells (c), Cytokeratin marker, Ck-MNF116 shows epithelial staining (d), carcino-embryonic antigen, CEA is an oncofetal antigen expressed by some tumors but not in normal adult tissues (e), androgen receptor, AR is a predictive marker for choice of therapy (f), muscle specific actin, MSA identifies the stromal component (g), and Ki-67 is a cell proliferation marker used for quantifying the proliferative index of the tumor (h). Scale bar: 100 µm.

A primary tracheal subocclusive mucoepidermoid tumor specimen was obtained from a 6-year-old girl who underwent segmental tracheal resection and primary surgical reconstruction. The excised part of the trachea measured 1.4 cm in length and 1.5 cm in width. In the lumen, a 1.3 by 1.2 by 0.9 cm polypous tumor was present. The resection margins of the tumor were inked before gross examination. Transversal sections from cranial to caudal parts were made and subsequent Haematoxylin Eosin (HE) staining revealed a polypoid tumor partly covered with respiratory mucosa (Figure 1a). Evaluations after periodic acid-Schiff staining and immunohistochemical labeling with antibodies against Muc-1 showed that the tumor was solid with trabecular/insular growth patterns and cystic with mucus-secreting cells (Figure 1b and c). Further, there were foci with squamous differentiation without atypia in desmoplastic stroma with psammoma calcifications. The tumor was confined to the mucosa and submucosa with no signs of expansion into the tracheal cartilage. No necrotic areas or marks of vascular/perineural invasion were detected, but various degrees of chronic inflammation were noted in the tracheal mucosa. In addition, antibodies against the cytokeratin marker Ck-MNF116 stained epithelial cells (Figure 1d), as did antibodies against carcino-embryonic antigen (CEA) (Figure 1e). Androgen receptor (AR) labeling was negative (Figure 1f). The stromal component in the tumor cells was negative for smooth muscle actin (SMA) (Figure 1g). Immunostaining with the cell proliferation marker Ki67 demonstrated that few mitoses were present. The tumor cells had a proliferation index of 5% on average, with a few hot spots reaching a proliferation index of up to 10% (Figure 1h). With PCR-analysis, we identified positive fusion transcript CRTC1-MAML2, which is associated with translocation t(11;19) (q21;p13) [8], [38]. Taken together, and due to the rarity of the diagnosis, there are different ways to classify the grade of the tumor. According to the NCCN guidelines for mucoepidermoid tumors, the patient presented a low-grade tumor classification of T1N0M0. This is also compatible with a tumor diagnosis of low (according to AFIP/Auclair) [39] and intermediary (according to Brandwein) grade [40].

### Isolation and expansion of mucoepidermoid tumor cells sorted for MSC markers

**Figure 2:**
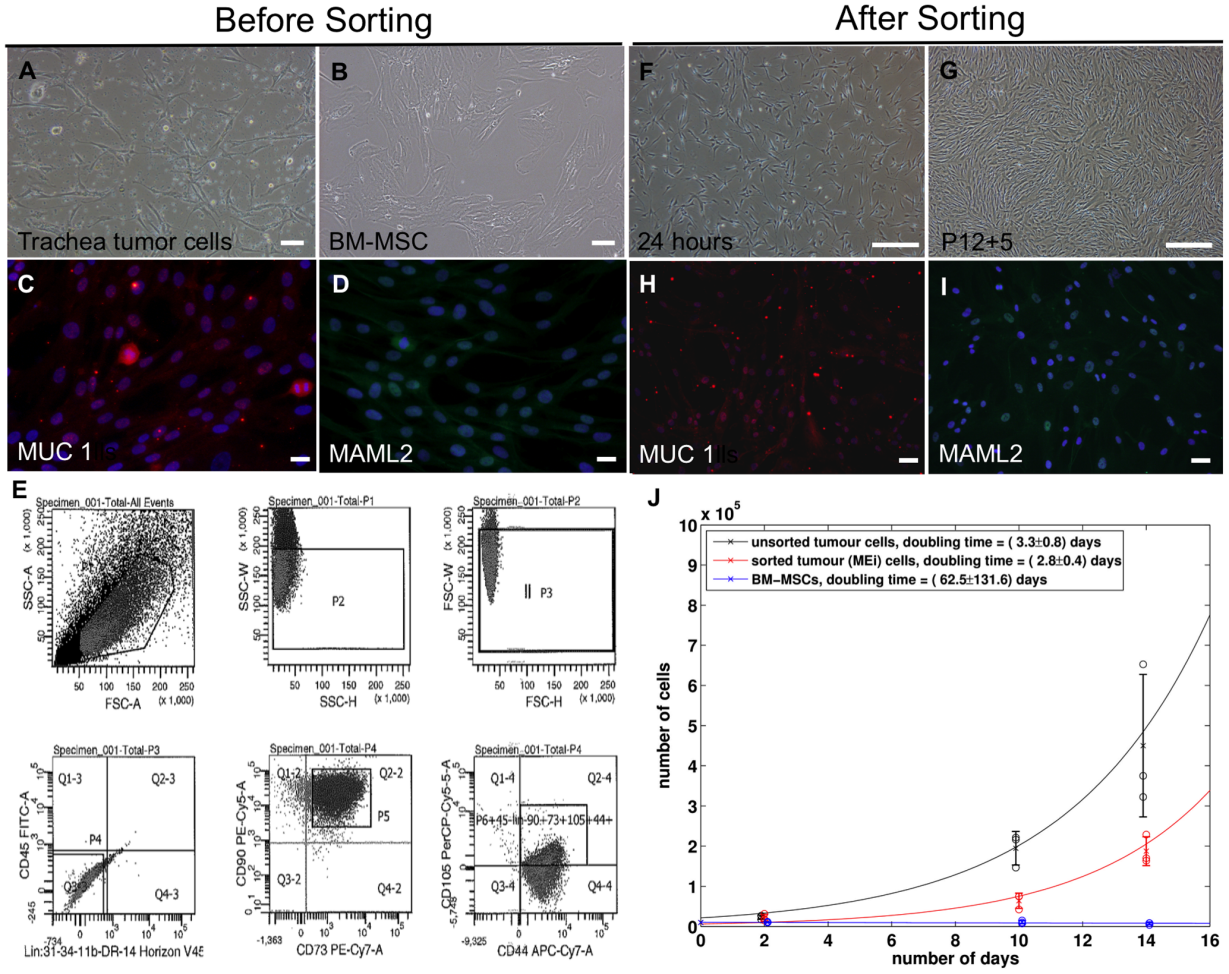
Cell morphology, lineage tracing, FACS sort and proliferation rates of mucoepidermoid tumor before and after sorting. Mucoepidermoid tumor cells at passage 0, Scale bar: 100 µm (a), BM-MSCs at passage 6, Scale bar: 100 µm (b), a representation of the merged images of MUC 1 and MAML2 before, Scale bar: 20 µm (c, d) and after, Scale bar: 50 µm (h, i) sort, flow cytometry sorting for a panel of mesenchymal stromal cell markers (e), post-sorting of MEi cells after 24 hours recovery, Scale bar: 500 µm (f) and a representative image of expanded post-sorted p12+5 MEi cells, Scale bar: 500 µm (g), cell proliferation rates and doubling times of unsorted and sorted cells (black and red respectively) *versus* BM-MSC (blue) (j). Colored circle indicates the raw datasets for the number of cells in each well; crosses and error bars indicate means and standard deviations.

For cell culture, tumor tissue was homogenized and seeded as described in *Materials and Methods*. Monolayer cultures yielded cells with spindle-shaped fibroblast-like appearances, closely resembling bone marrow-derived mesenchymal stromal cells (BM-MSCs) (Figure 2a and b). Mucoepidermoid tumors arise from the serous and mucous glands of the upper airway and salivary glands. With a possibility that the tumor cells originate from mesenchymal stromal cells, we aimed to investigate differences and similarities between tracheal tumor cells and BM-MSCs. To characterize the expanded mucoepidermoid tumor cells, we confirmed the tumor lineage trace by MUC 1 and MAML2 expression before (passage 3; Figure 2c and d) and after (passage 12+5; Figure 2 h and i) cell sort. We found the majority of cells expressed MUC 1 and MAML2 (89% and 95% respectively). By fluorescence-activated cell sorting (FACS) analyses, we detected a nearly homogenous phenotype displaying typical MSC characteristics: 98% strongly expressed CD44, CD73, CD90 and CD105, and were negative for haematopoietic markers CD11b, CD14, CD34, CD45 (Figure 2e). The post-sorted mucoepidermoid tumor cells maintained their morphology throughout the expansion process up to passage 12+6 and will hereafter be designated as MEi (MSC-like mucoepidermoid tumor) cells (Figure 2f and g). We noted that the doubling time of unsorted tumor cells (3.3±0.8 days) and MEi cells (2.8±0.4 days) were considerably faster than BM-MSCs (63±132 days). Using an unpaired *t*-test, we determined that there was no significant difference between the proliferation rates of unsorted tumor cells and MEi cells (see experimental procedures). Clonogenic assay also demonstrated that MEi cells (123±38.97) contained more CFU-F colonies in all triplicates when compared to BM-MSCs (53.33±2.52).

### *In vitro* differentiation of MSC-like mucoepidermoid tumor (MEi) cells

**Figure 3:**
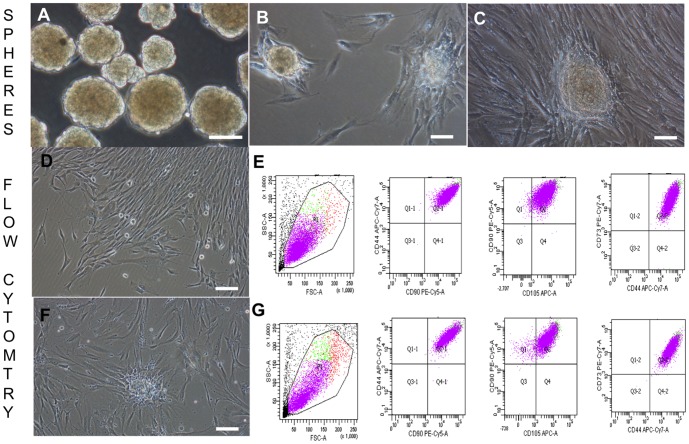
Spheroid cultures and flow cytometry analyses of spheroid outgrowths. Day 10 spheroids in low adhesion culture plates (a), day 10 trachea spheroids plated onto culture plates after 24 hours (b), outgrowths from spheroid cultures (c). Flow cytometry analyses of spheroid outgrowths after 25 days *in vitro*. Scale bar: 100 µm. Cell morphology before (d) and after (f) trypsinization. Scale bar: 200 µm. Flow cytometry analyses for a panel of mesenchymal stromal cell markers before (e) and after (g) trypsinization.

**Figure 4:**
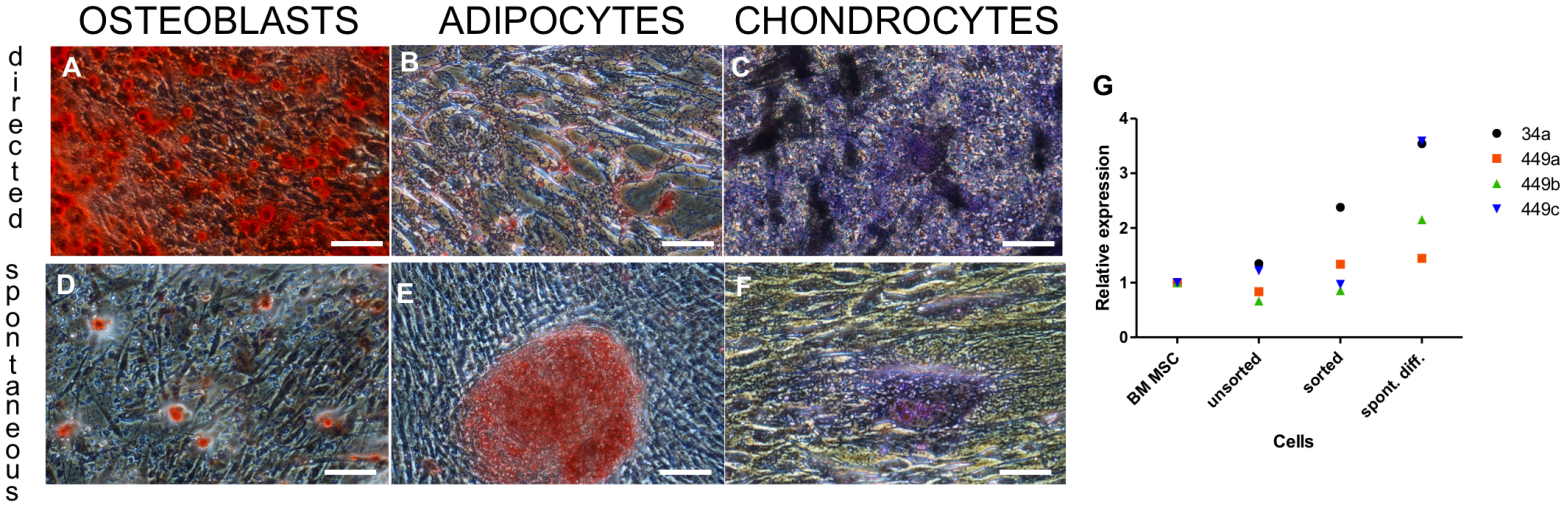
Differentiation of MEi (MSC-like mucoepidermoid tumor) cells to mesenchymal trilineage. Directed differentiation with growth factors (a–c): towards osteoblast phenotype (alizarin Red staining) (a), towards adipocyte phenotype (Oil red staining) (b), towards chondrocyte phenotype (toluidine blue staining) (c); Spontaneous differentiation without growth factor (d–f). alizarin S staining (d) oil red staining (e), toluidine blue staining (f). Scale bars: 100 µm. MicroRNA analyses of miR-34, miR 449a, b, c for BM-MSC, unsorted, sorted and spontaneous differentiation of tracheal tumor cells (g).

**Figure 5:**
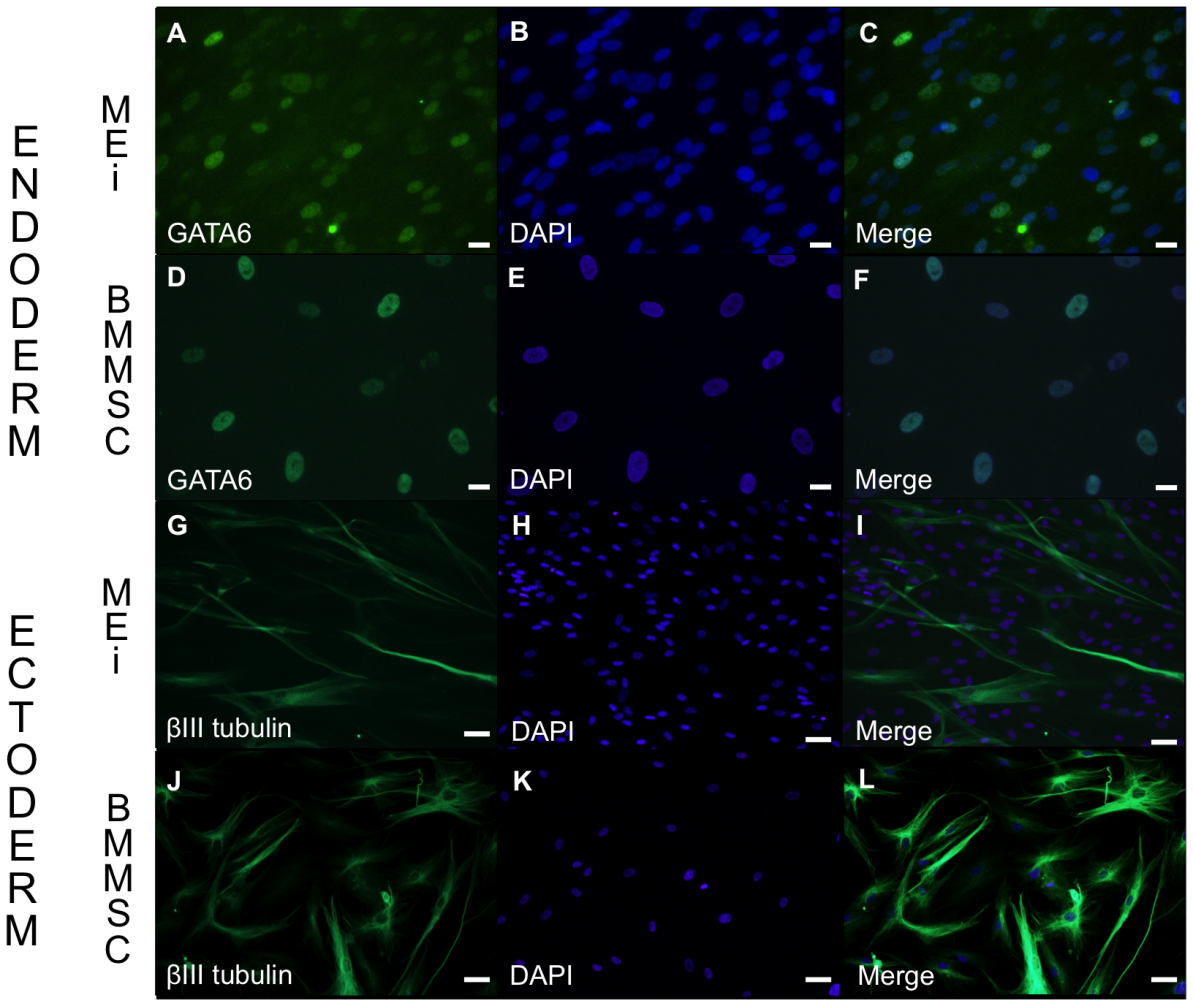
*In vitro* differentiation of MEi (MSC-like mucoepidermoid tumor) cells to endoderm and ectoderm lineages. A representative image of endoderm differentiation of MEi cells (a–c) and BM-MSCs (d–f) were stained positive for GATA 6, and ectoderm differentiation of MEi cells (g–i) and BM-MSCs (j–l) were positive for β-III tubulin. Scale bar: 50 µm.

Following trypsinization of MEi cells from passages 9+1, 12+2 and 12+6, we evaluated cell stemness by sphere formation and differentiation [41]. Spheroid bodies formed at an efficiency rate close to 100% in suspension cultures or in hanging drops under static culture conditions. Spheroids were compact by day 10 of culture, and had diameters ranging between 100 and 125 µm with a circumferential outer layer of endodermal-like cells (Figure 3a). When plated on organ culture tissue plates such spheroid bodies' outgrowths could be further cultured for up to 1 month (Figure 3b and c). However, after 25 days, 99.9% of 10,000 counted cells had retained a pronounced mesenchymal stromal cell morphology and immuno-phenotype, which may typically be the long-term dormant cells (Figure 3d–g). To investigate their differentiation capacity, passage 12+3 MEi cells were cultured in three types of defined growth factor-enriched medium (see experimental procedures). In each appropriate medium, 80% of cells differentiated either towards osteoblast (Alizarin R staining; Figure 4a), adipocyte (Oil red staining; Figure 4b) or chondrocyte lineages (Toluidine blue staining; Figure 4c). To study spontaneous differentiation, cells were grown in standard culture media devoid of growth factor enrichment. We found that approximately 10% of the cells spontaneously differentiated towards the three phenotypes *i.e.* osteoblast, adipocyte and chondrocyte (Figure 4d–f). Although the *in vitro* differentiation potential evaluations proved that MEi cells are multipotent, microRNA biomarkers miR 34/449 family was observed to be differentially expressed in the tracheal tumor cells when compared to the BM-MSCs (Figure 4g). We further explored the differentiation potential of MEi cells to both endoderm and ectoderm lineages in comparison to BM-MSCs (Figure 5). We observed that a subpopulation of MEi cells were positive for GATA6 (Figure 5a–c) and β-III tubulin (Figure 5g–i) (51% and 12.5% respectively) were similar to BM-MSC (Figure 5 d–f, j–l)

### Differences in gene expression profiles between MEi cells and BM-MSCs

**Figure 6:**
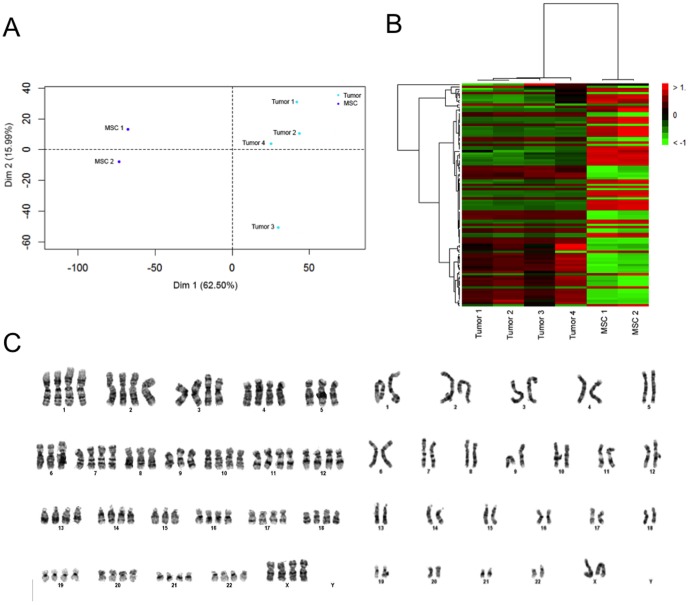
Gene expression profiles and karyotyping of MEi cells. Two-dimensional cluster plots for the classification of samples based on the first two principal components. Tumor 1, 2, 3, 4 are MEi cells and MSC 1, 2 are BM-MSCs (a), Each row represents a gene and each column represents a sample. The expression level of each gene in a single sample is relative to its median abundance across all samples and is depicted according to a color scale shown on the right. Red and green indicate expression levels respectively above and below the median. (b), a representative image of tetraploidy and normal karyotype (c) at passage 12+3.

To investigate potential differences between MEi cells and BM-MSCs, we assessed their respective gene expression profiles using microarray analysis. When examined with Principal Components Analysis (Figure 6a) as well as through hierarchical clustering (Figure 6b), MEi cells and BM-MSCs formed distinct constellations that were clearly separated from each other. These results indicate that the MEi cells are different from the BM-MSCs based on their gene expression profile patterns. Using unpaired *t*-test, we found that a total of 1900 genes were significantly different between MEi cells and BM-MSCs (p<0.02). The MEi cells were found to not display gene expression profiles that were associated with normal stem or tumorigenic cells such as SOX2, OCT4, C-MYC.

To further understand the biological differences between MEi cells and BM-MSCs, enrichment analysis using the Database for Annotation, Visualization and Integrated Discovery (DAVID) Gene Functional Classification Tool was performed using the differentially expressed genes as input. From the full list of pathways in the Kyoto Encyclopedia of Genes and Genomes (KEGG) database, pathways in cancer, purine metabolism and ECM receptor interactions were found to significantly enriched by the differentially expressed genes (Table 1). Thus, it is likely that these pathways contribute to the functional differences that were observed between MEi cells and BM-MSCs. For the KEGG annotation ‘Pathways in cancer’, a partial list of the differentially expressed genes most relevant to this pathway is provided in Table 2. Interestingly, genes that have been previously found to be overexpressed in other cancers such as hepatocyte growth factor (HGF), laminin alpha 4 (LAMA4) and androgen receptor (AR) were also found to be more highly expressed in MEi cells compared to BM-MSCs.

**Table 1:** KEGG pathways significantly enriched by genes differentially expressed between MEi cells and BM-MSCs.

KEGG Pathway	Count	%	P-Value	Fold Enrichment
hsa05200: Pathways in cancer	21	4.95283	1.33E-04	2.566784657
hsa00230: Purine metabolism	12	2.830189	0.001337	3.134784623
hsa04810: Regulation of actin cytoskeleton	14	3.301887	0.002217	2.627037817
hsa04512: ECM-receptor interaction	8	1.886792	0.004564	3.806524184
hsa04510: Focal adhesion	12	2.830189	0.00966	2.422333572

Pathways were identified by DAVID Functional Annotation and ranked by P-value with a cutoff of 0.01. Counts and percentages refer to the number and percentage of genes from the input list that fit into a given KEGG pathway. Fold enrichment is the magnitude of enrichment for each KEGG pathway compared with the entire gene list in the Affymetrix Human Gene 1.1 ST Array that serves as the reference.

**Table 2:** Partial list of differentially expressed genes from the KEGG annotation: Pathways in cancer.

Gene Symbol	Gene Name	Log_2_FC	adj. P-Value
Hgf	hepatocyte growth factor (hepapoietin A; scatter factor)	−4.25008	0.000379
Mmp1	matrix metallopeptidase 1 (interstitial collagenase)	−4.19169	2.19E-05
PPARG	peroxisome proliferator-activated receptor gamma	−3.72941	1.02E-05
LAMA4	laminin, alpha 4	−3.60148	0.003373
KITLG	KIT ligand	−3.57558	0.000572
MAPK10	mitogen-activated protein kinase 10	−3.00792	0.013107
WNT5A	wingless-type MMTV integration site family, member 5A	−2.97522	2.19E-05
Ar	androgen receptor	−2.62261	0.000197
MECOM	ecotropic viral integration site 1	−2.60297	0.000109
Lama2	laminin, alpha 2	−2.26819	0.008147
DAPK2	death-associated protein kinase 2	−2.19753	0.000183
cdkn2b	cyclin-dependent kinase inhibitor 2B (p15, inhibits CDK4)	2.299025	0.000169
TGFB2	transforming growth factor, beta 2	4.278179	3.95E-05

Log_2_FC is the base 2 logarithm of the fold change, with negative values indicating that the gene is more highly expressed in the tumor samples.

Functional Annotation Clustering groups all the Gene Ontology (GO) terms that are significantly enriched in order to reduce redundancy, as many of the GO terms are similar. When the Functional Annotation Clustering of GO terms was performed using the differentially expressed genes as input, several interesting clusters with high enrichment scores were obtained. These clusters represent those that are related to tube development, cell adhesion, angiogenesis, signal transduction, inflammatory response and regulation of programmed cell death, suggesting that these areas contribute to the differences observed between MEi cells and BM-MSCs. A partial list of the differentially expressed genes found in the GO category ‘Tube development’ is shown in Table 3. Taken together, our results show that the gene expression differences between trachea tumor cells and BM-MSCs were related to the origin and tumorigenicity of MEi cells.

**Table 3:** Partial list of differentially expressed genes from the Gene Ontology term: Tube development.

Gene Symbol	Gene Name	Log_2_FC	adj.P.Val
Tcf21	transcription factor 21	−5.24902	7.07E-05
tbx5	T-box 5	−4.87505	4.59E-06
FOXF1	forkhead box F1	−3.75911	4.04E-05
FOXP2	forkhead box P2	−2.90287	0.000955
PBX1	pre-B-cell leukemia homeobox 1	−2.72587	0.000128
tbx3	T-box 3	−2.71177	0.000491
EDNRA	endothelin receptor type A	−2.1	0.005303
Plxnd1	plexin D1	2.479673	0.000134
RBP4	retinol binding protein 4, plasma	2.808411	3.58E-05
PDGFA	platelet-derived growth factor alpha polypeptide	2.861353	0.000571
fgf1	fibroblast growth factor 1 (acidic)	3.334209	0.0053
alx1	ALX homeobox 1	3.370444	4.57E-05
nog	noggin	3.516299	1.61E-05

Log_2_FC is the base 2 logarithm of the fold change, with negative values indicating that the gene is more highly expressed in the tumor samples.

### Genome characterization of MEi cells

To investigate the chromosomal stability and copy number variations of the post-sorted, expanded MEi cells, karyotyping and comparative genomic hybridization (CGH) analyses were performed. MEi cells were passaged three times prior to treatment overnight with Colcemid at 37°C to synchronize the cell cycle to metaphase. After fixation, cells were stained and 100 metaphase spreads (50 per sample) underwent karyotype analyses. We found that the expanded cultures of sorted cells were mostly normal; 46XX but noticed that 9 out of 50 (sample 1); 12 out of 50 (sample 2) counts showed tetraploidy with 85 to 92 chromosomes (Figure 6c). CGH with an 180K oligo array further revealed several recurrent benign copy number variations. In addition, a small duplication of unknown significance was detected on chromosome X, Xq21.31, ∼156 kb (min region chrX: 87,695,049–87,851,109 in hg19). The region did not contain any known gene and the variant was interpreted as a likely benign variant. These evaluations confirm that the majority of the multipotent MEi cells possessed a normal karyotype.

### MEi cells did not form teratomas

Teratoma formation assay is commonly used in tumor cell biology to determine cell potency. We therefore injected MEi cells (passage 18 at a dose of 2×10^6^/animal) into testis or subcutaneous tissue in severely compromised immunodeficient (SCID-Beige) mice. Ocular inspection and palpation after eight weeks revealed no discernible signs of *in vivo* growth in any of the 6 and 5 animals. Further, microscopical analyses (FISH analysis using a probe specific for human X/Y chromosomes) were negative; indicating that MEi cells did not propagate *in vivo*.

## Discussion

‘Adult’ stem cells (or better denoted ‘tissue specific’ stem cells) are believed to maintain tissue homeostasis during injury but could, if become dysfunctional also provoke benign or malignant tumors according to the cancer stem cell paradigm [42]. To date, tissue specific stem cells have not been demonstrated in transformed tissues from the human upper respiratory tract.

In the present study, we identified and expanded mesenchymal stem cell-like (MEi) cells in primary cultures from a rare benign paediatric mucoepidermoid tracheal tumor. We validated the tumor origin by immunocytochemistry and revealed high expression for MUC 1, MAML2 and an absence for smooth muscle actin [6]. While tetraploidy is not uncommon in healthy subjects, it is usually not as high as the 20% observed [43]. Furthermore, balanced translocations are not detected by array CGH. Hence, it is still plausible that the mucoepidermoid tumor did not include cross-contamination artifact from mesenchymal cells that migrated from the healthy tracheal tissue region surrounding the tumor location.

We report that the self-renewal and differentiation potential of MEi cells had overlapping properties to normal BM-MSC: *(i)* obtaining a nearly homogenous immuno-phenotype displaying typical MSC characteristics, *(ii)* lacked *in vivo* tumor initiation capacity, *(iii)* demonstrated fibroblastoid colony forming ability and *(iv)* retained mesenchymal tri-lineage differentiation capacity *i.e.* osteoblasts, adipocytes and chondrocytes with and without growth factor induction. In contrast, there were distinct cellular differences noted between MEi cells and BM-MSCs [44]. The cell proliferation rates of MEi cells between the early (passage 3) or late passages (passage 18) were exponentially increasing in cell numbers.

The gene expression profiling showed however distinct differences between MEi cells and BM-MSCs with regard to genes relating to tube development, cell adhesion, angiogenesis, signal transduction, inflammatory response and regulation of programmed cell death. Unlike BM-MSCs, the MEi cells did not show expression of the Androgen receptor when tested by immunohistochemistry, but more sensitive microarrays found a 4-fold up-regulation of RNA when compared to BM-MSCs.

The initial small size of the tumor biopsy did not easily allow for analysis of the frequency of MEi cells in the original sample. For this we instead developed a mathematical model from which we estimate a frequency of 17.5% MEi cells in the mucoepidermoid tumor cell mass. Intriguingly, we found the mucoepidermoid tumor cell mass may possibly contain niche cells (*i.e.* resident stem cells/quiescent stem cells). Immunocytochemistry staining of MEi cells revealed a small sub-population of MEI cells; 12.7% expressed β-III tubulin (ectodermal marker) and 49.4% expressed GATA 6 (endodermal marker) were differentiating similarly like BM-MSCs. Assuming that the niche cells expressed both markers, the percentage of MEi cells that are niche cells is 6.3%. Given that 17.5% of the tumor cells are MEi cells, the percentage of tumor cells that are niche cells is therefore 1%.

To our knowledge, the present study is the first report demonstrating a distinct population of benign human tracheal tumor cells with stem cell-like properties and warrants further studies on the role of these cells in initiation, development and/or progression of this type of tumor of the upper respiratory tract.
