# Expression of Concern: Characterization of Stem-Like Cells in Mucoepidermoid Tracheal Paediatric Tumor

**DOI:** 10.1371/journal.pone.0313109

**Published:** 2024-10-28

**Authors:** The PLOS ONE Editors

After this article [1] was published, questions were raised about whether there was adequate evidence to support claims about the nature of cells used in the study; about the reliability of results for which supporting data were not reported in the article; and about the source(s) and ethics approval(s) in place to support work with human bone marrow-derived mesenchymal stromal cells (BM-MSCs). PLOS followed up on these issues in accordance with Committee on Publication Ethics (COPE) guidance. The corresponding author could not be reached in these post-publication discussions, but co-authors provided some supporting data and clarifications as outlined below.

## Evidence to support claims that cells used in this study were cancer cells, and that there are specialized stem cell-like cells in the tumor

A member of *PLOS ONE*’s Editorial Board advised that PCR and karyotype analysis data would be needed to support claims about the nature of the cells used in the study, and that without these data the article’s conclusions are not adequately supported.

PCR data were not provided to confirm the t(11;19) (q21;p13) translocation in the primary tumor cells, and the PCR method and reagents used were not reported in sufficient detail to enable replication. The authors noted that the tumor diagnosis was provided by pathologists, and that information on the mutation was reported in the article as background information about the materials used but was not intended as a primary result of the reported study. The authors also commented that PCR analysis to confirm the translocation was performed by the Department of Pathology and Cytology, Karolinska University Hospital, and that the dataset is retrievable from the hospitals archived pathological-anatomical diagnosis data repository but cannot be made publicly available due to patient privacy considerations.

Questions were raised about differences between PCR results obtained for the primary tumor and the karyotype of MEi cells isolated from that tumor. The authors commented that this may be due to genetic drift. The consulted member of the Editorial Board advised that they were satisfied with this response.

The authors provided image data (S1 File and S2 File) to support the statement, "microscopical analyses (FISH analysis using a probe specific for human X/Y chromosomes) were negative; indicating that MEi cells did not propagate in vivo." They also stated that negative FISH findings cannot prove a lack of cellular growth other than in the particular section studied, and noted that the statement, “Mei cells did not form teratomas,” was based on visual inspection, palpation, and FISH analysis.

## Controls and replication

Concerns were raised about insufficient replication within the study, and about the generalizability and overall reliability of the results and conclusions since the study relied on a single tumor from a single patient. These limitations of the study design were not discussed in the article [1].

In addition, questions were raised about the study’s controls. The study used BM-MSCs from healthy volunteers as positive controls, but did not include controls based on normal tissue from the patient who was the source of tumor cells. Also, control data were not reported in Figs 2–4, except in Fig 2B and 2J.

The authors did not respond to PLOS’ questions pertaining to controls and replication and so these issues remain unresolved. Nevertheless, the member of the Editorial Board advised that the conclusions are well-supported for an initial characterization study.

## Ethics

Karolinska Institutet confirmed that the authors had ethics approval for the animal and human subjects research reported in the article (permits S180-12 and N173-10 for animal research, 2008/307-31 and 2012/2163-31 for human subjects research).

BM-MSCs were obtained in the laboratory of Dr. Hong Qian (Karolinska Institutet) under ethical permit 2012/1971-31/3, approved by the local ethics committee at Karolinska Institutet. PLOS did not receive any information about whether the cells were validated or authenticated before the experiments reported in [1].

## Data

PLOS was notified that the Advanced Center for Translational Medicine, Department for Clinical Science, Intervention and Technology, Division of Ear, Nose, Throat at the Karolinska Institutet was shut down and all documentation and equipment were archived. The authors no longer have access to the original data for this study.

In light of these unresolved issues, the *PLOS ONE* Editors issue this Expression of Concern.

Note: The corresponding author is no longer available via the contact information listed on the article [1].

## Supporting information

S1 File Analysis of Mei-cell injected mice showing uncropped negative images for FISH X-chromosome signals. (ZIP)

S2 File Uncropped images of positive control (i.e. the expected parallel staining of mouse tissue with proven engrafted human tumor growth) showing that the lack of detectable signals in the experimental sections were likely due to lack of the specific ligand (i.e. lack of presence of X-chromosomes). (ZIP)
