# Supplementary figures and images for: Expression of Concern: Characterization of Stem-Like Cells in Mucoepidermoid Tracheal Paediatric Tumor
Source: PLoS One. 2024 Oct 28;19(10):e0313109. doi: 10.1371/journal.pone.0313109 (PMC11516170; doi:10.1371/journal.pone.0313109)

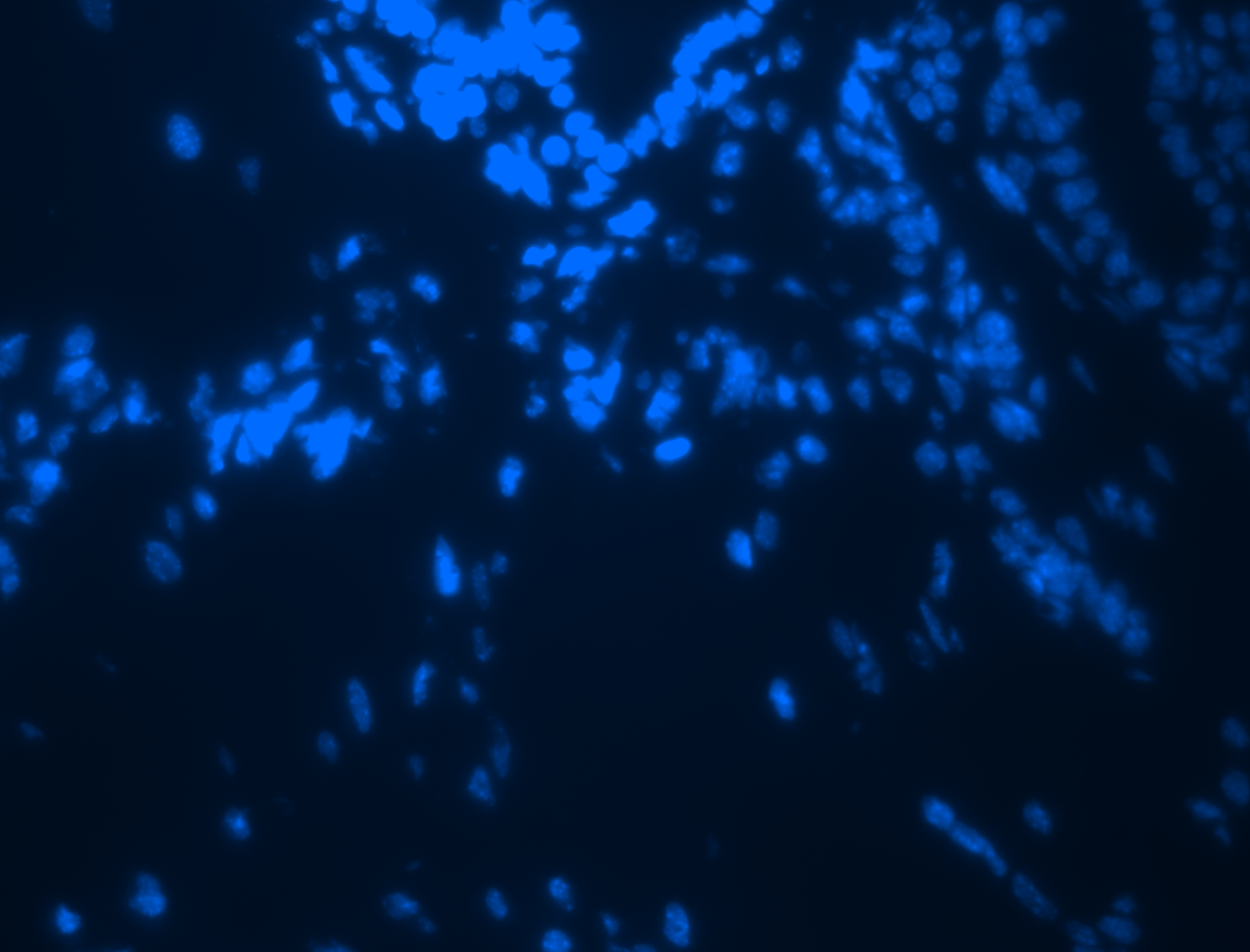

Supplement: S1 File — (ZIP) [file pone.0313109.s001.zip › neg area/Meil0055.tif]

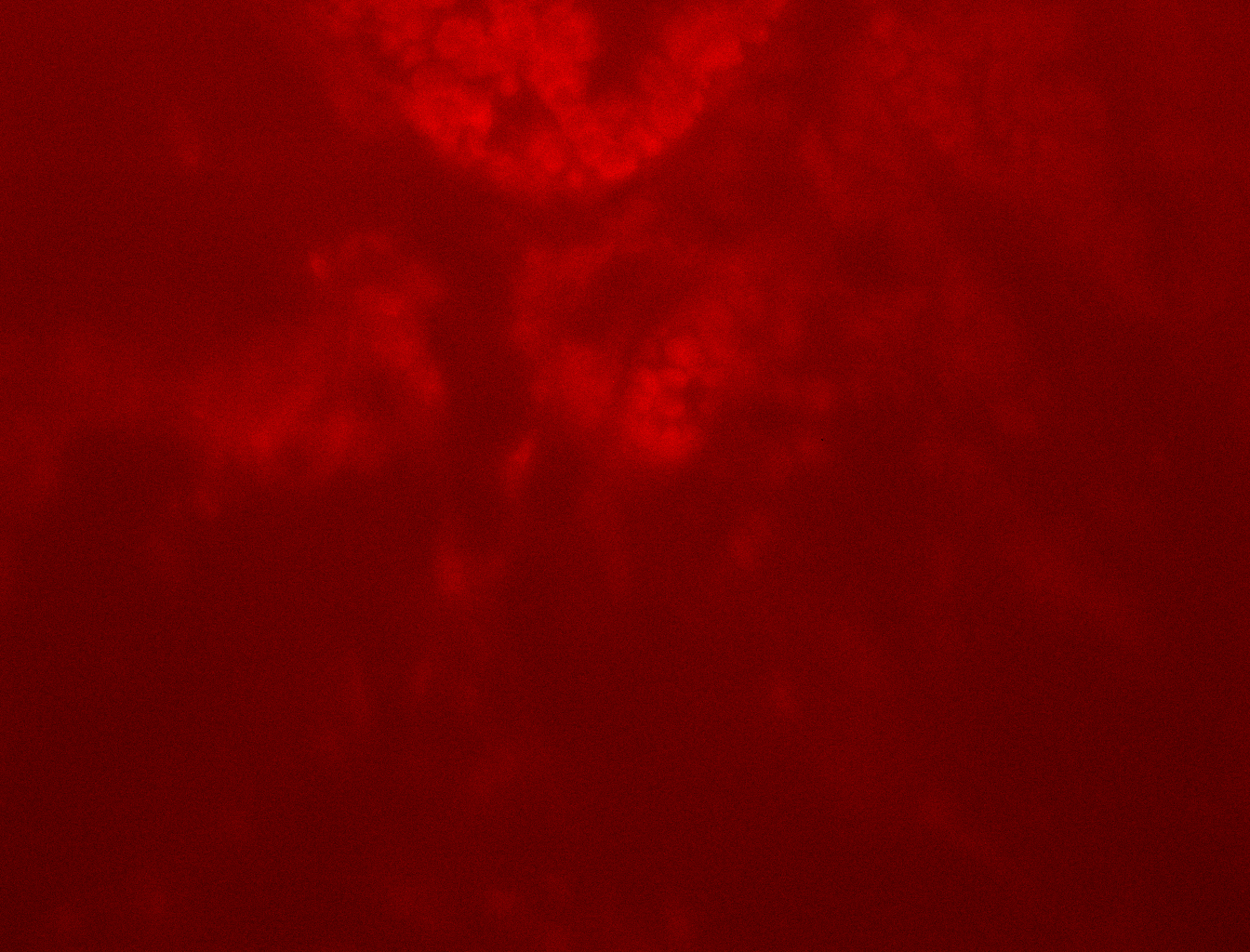

Supplement: S1 File — (ZIP) [file pone.0313109.s001.zip › neg area/Meil0057.tif]

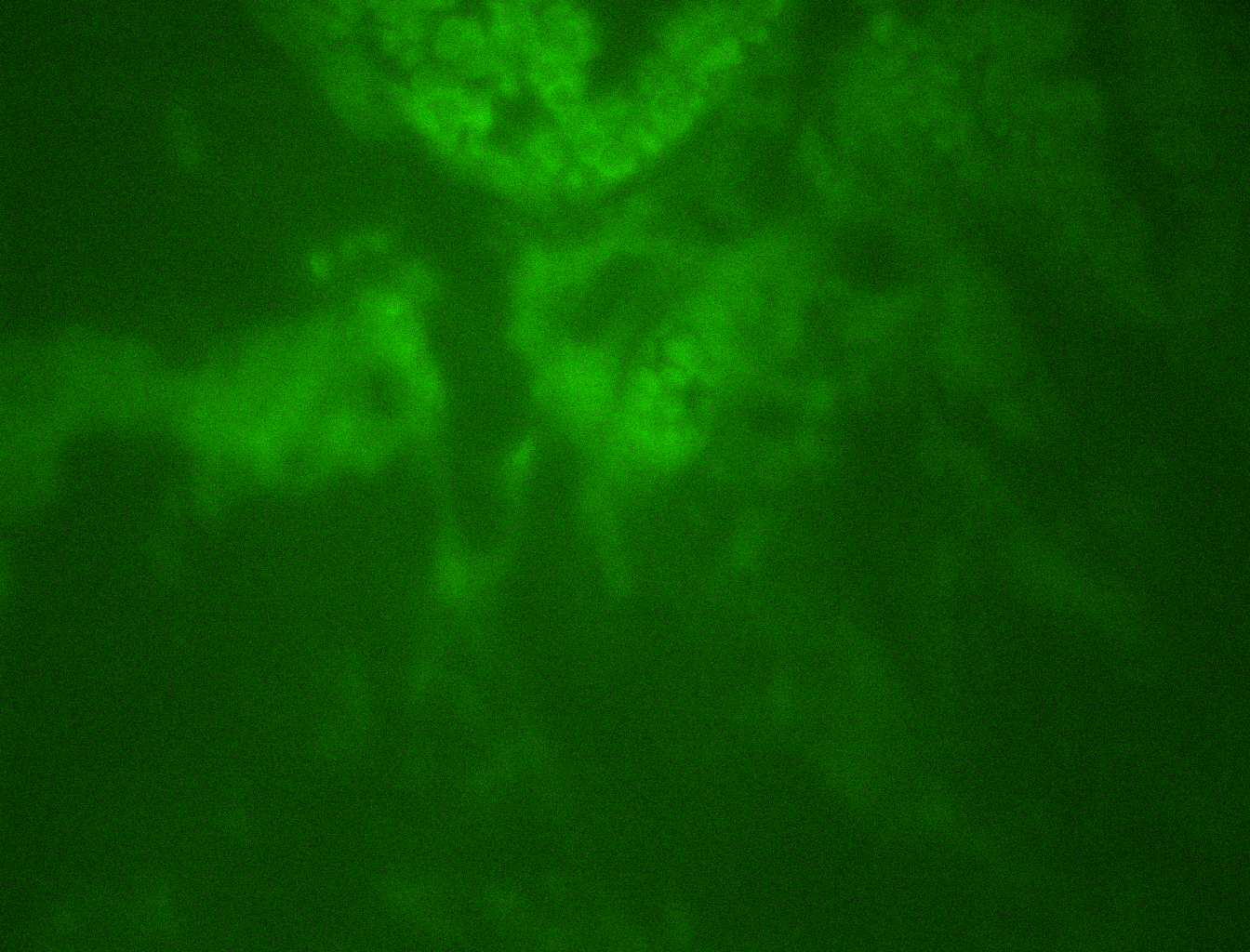

Supplement: S1 File — (ZIP) [file pone.0313109.s001.zip › neg area/Meil0056.tif]

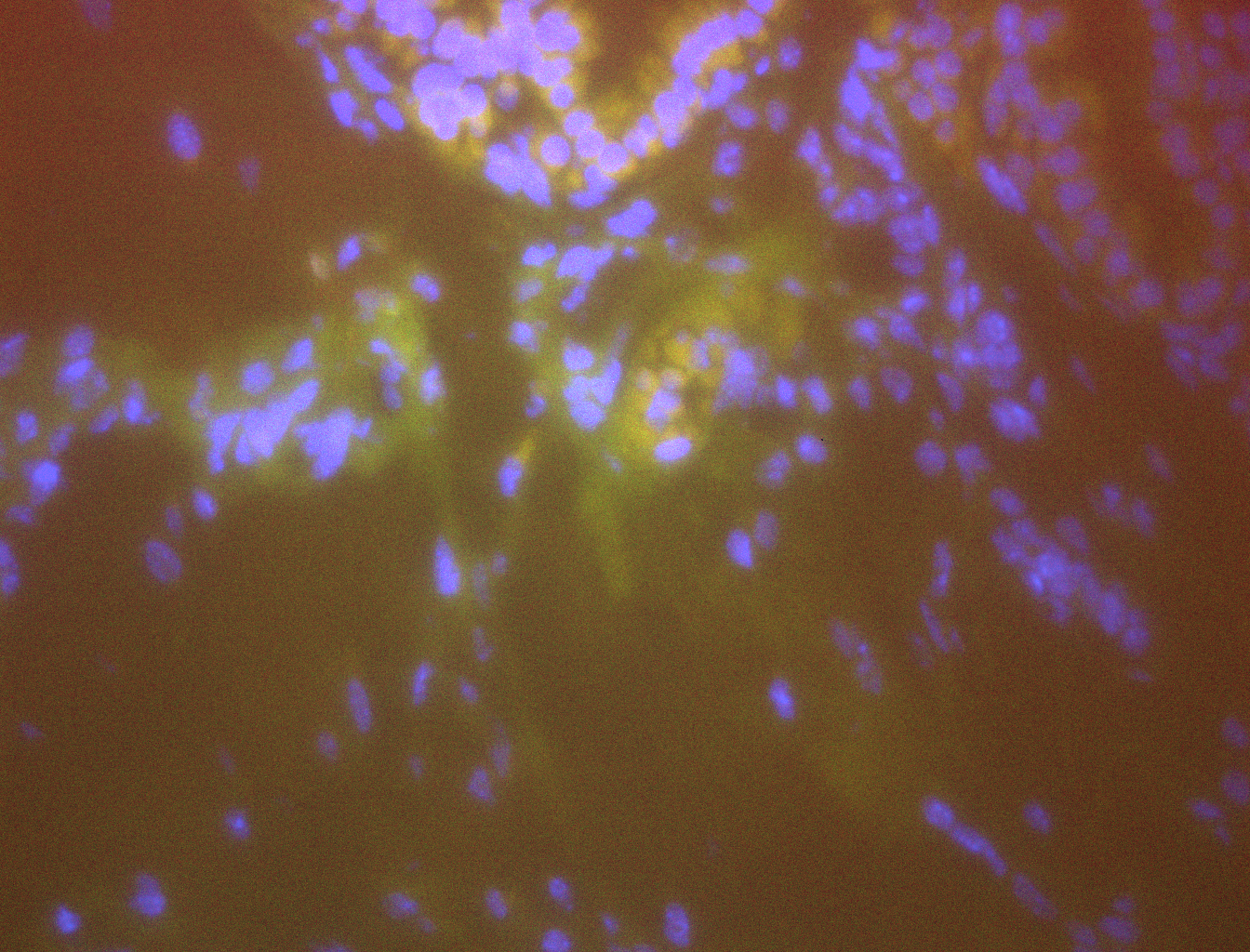

Supplement: S1 File — (ZIP) [file pone.0313109.s001.zip › neg area/Meil0058.tif]

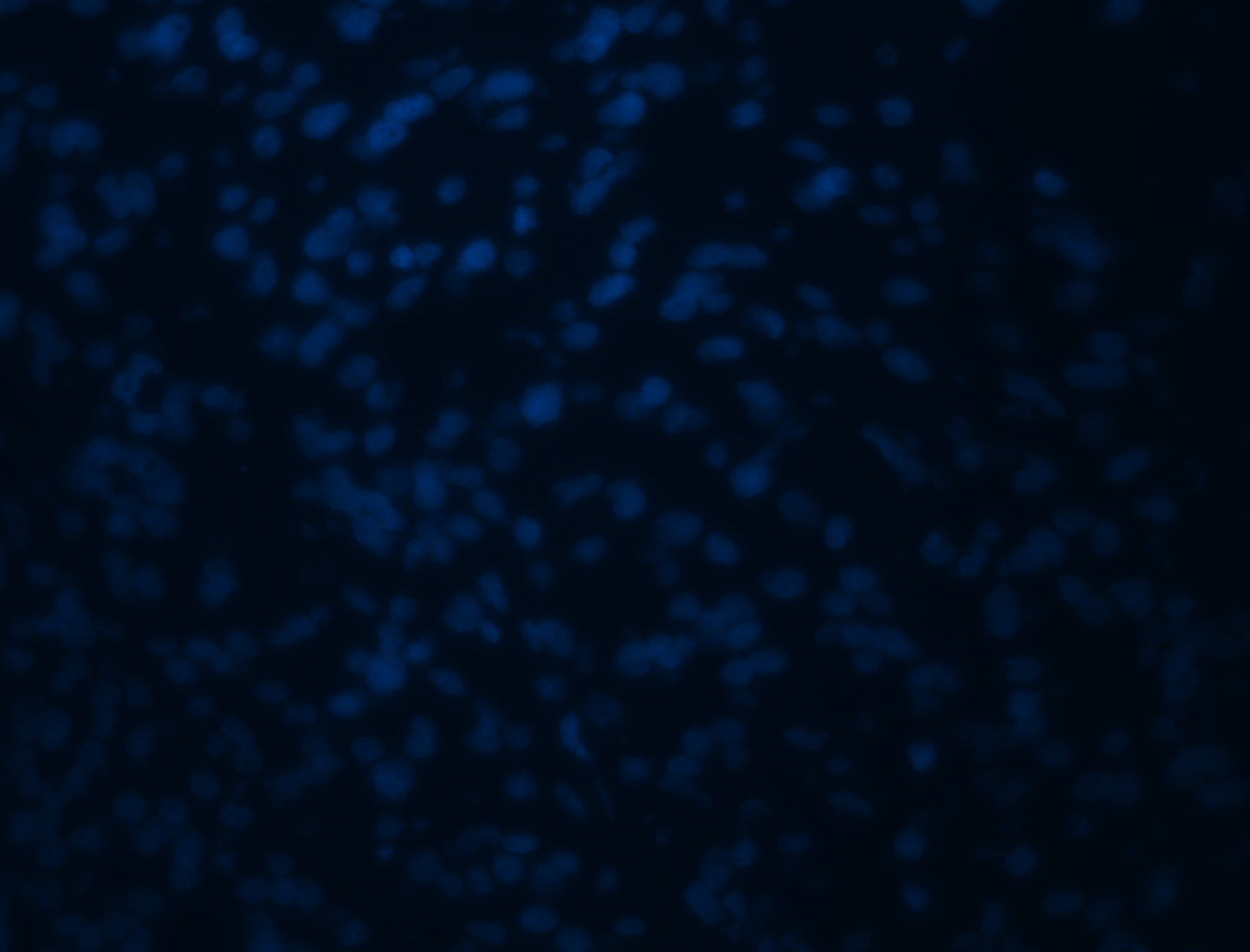

Supplement: S2 File — (ZIP) [file pone.0313109.s002.zip › pos ctrl/Meil0002.tif]

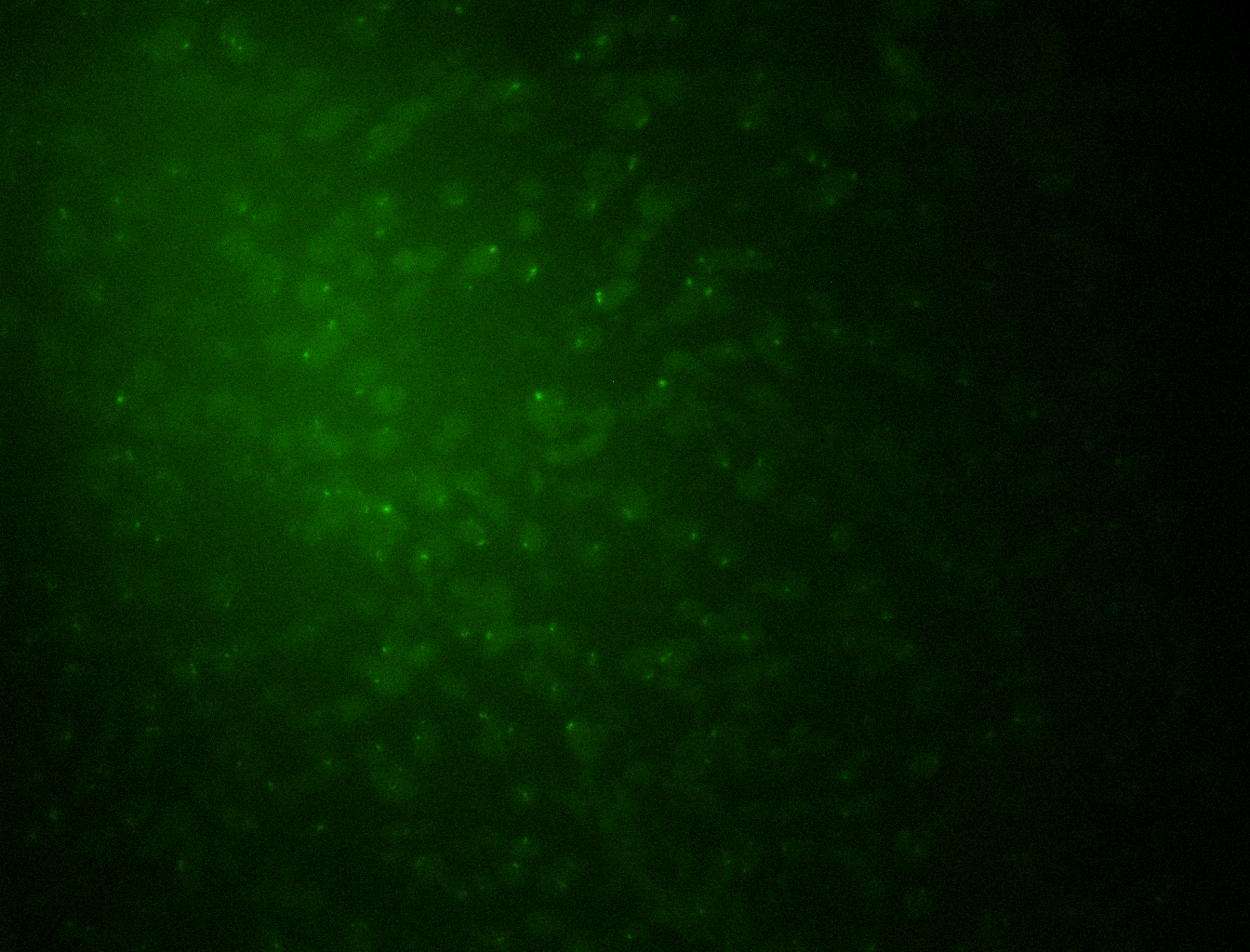

Supplement: S2 File — (ZIP) [file pone.0313109.s002.zip › pos ctrl/Meil0003.tif]

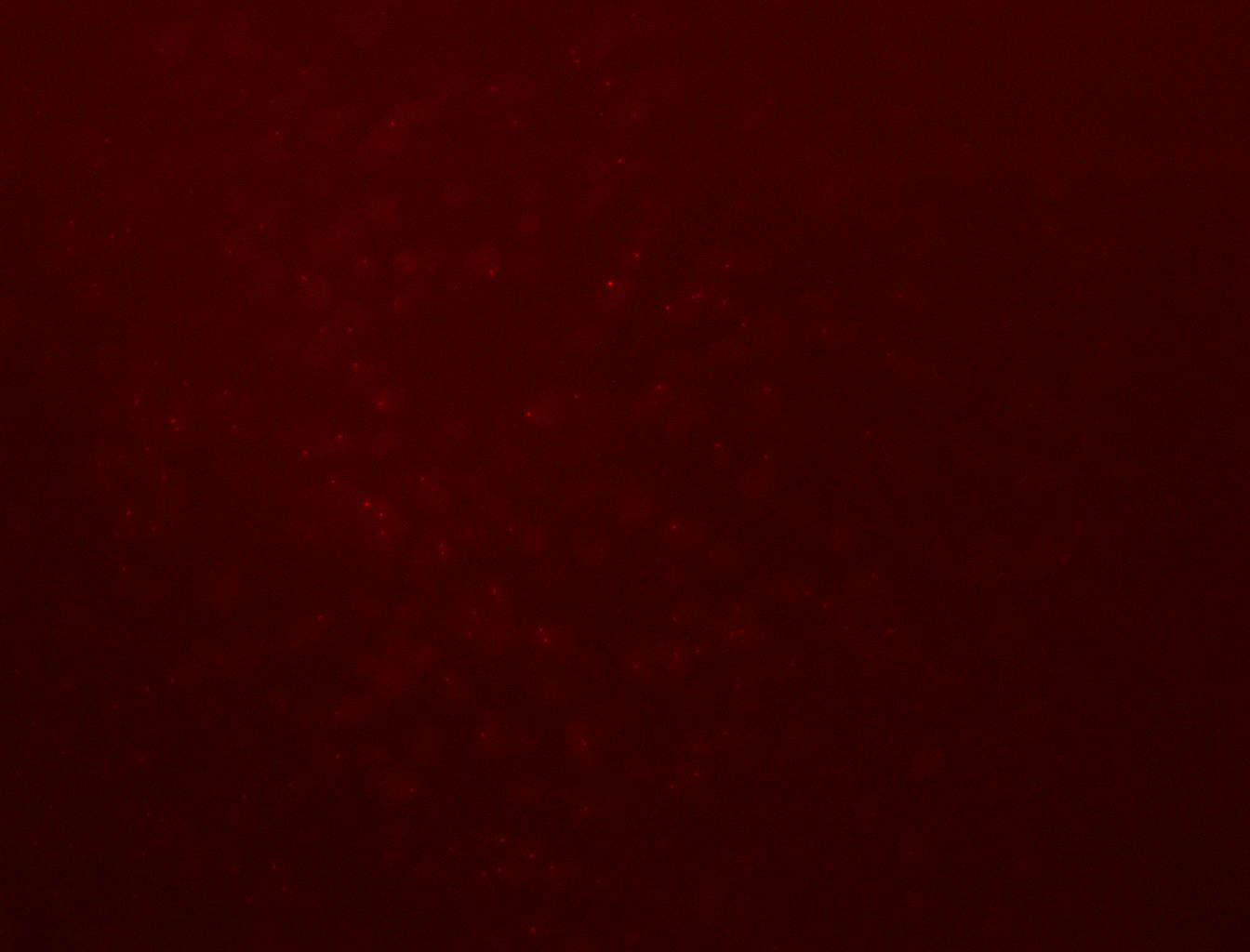

Supplement: S2 File — (ZIP) [file pone.0313109.s002.zip › pos ctrl/Meil0004.tif]

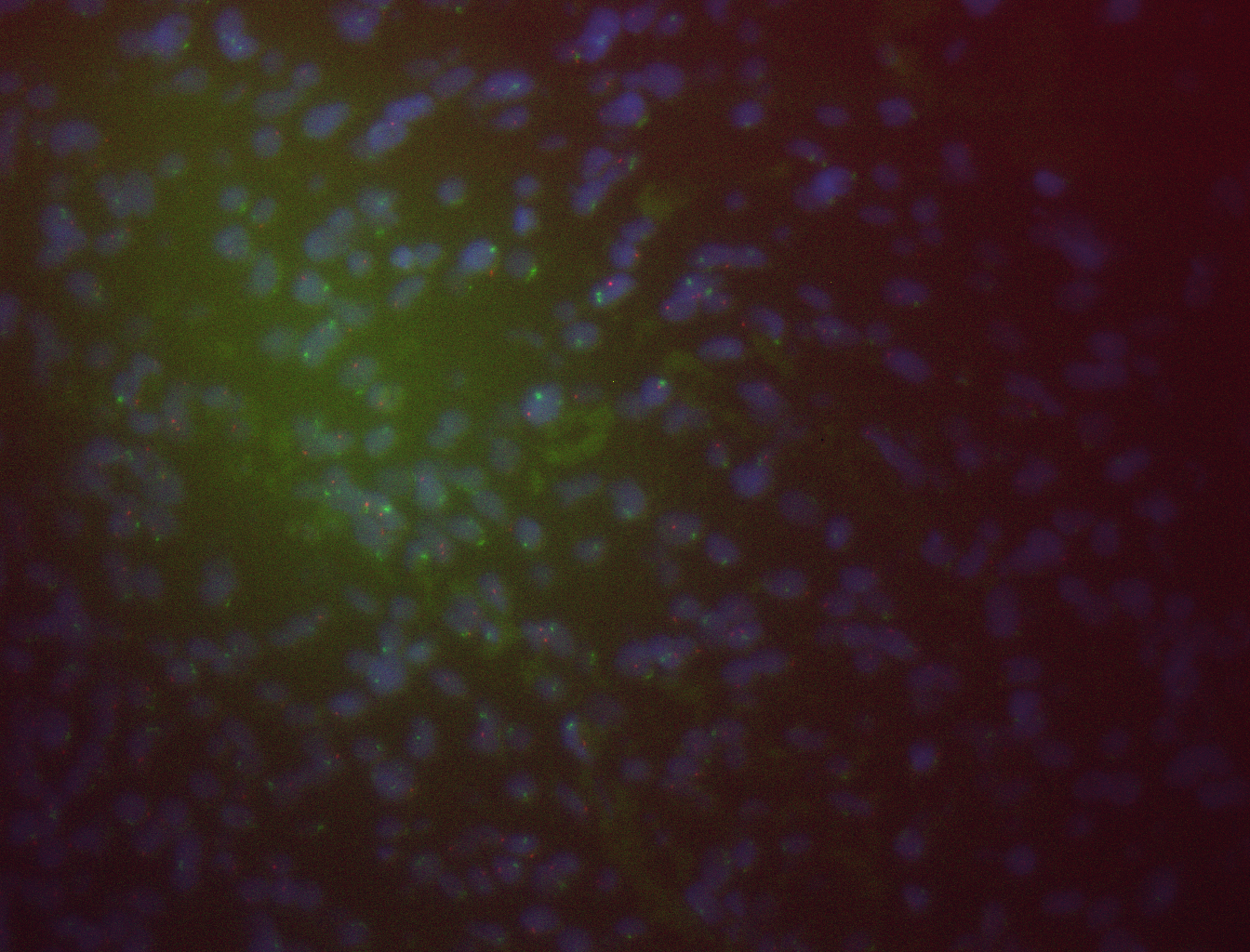

Supplement: S2 File — (ZIP) [file pone.0313109.s002.zip › pos ctrl/Meil0005.tif]
